# Patterns of clustering of six health-compromising behaviours in Saudi adolescents

**DOI:** 10.1186/1471-2458-14-1215

**Published:** 2014-11-25

**Authors:** Saeed G Alzahrani, Richard G Watt, Aubrey Sheiham, Maria Aresu, Georgios Tsakos

**Affiliations:** Public Health Department, Faculty of Medicine, Al-Imam University, P.O. Box 7544, Riyadh, 13317-4233 Saudi Arabia; Research Department of Epidemiology and Public Health, University College London, 1-19 Torrington Place, London, WC1E 6BT UK

**Keywords:** Health behaviours, Clustering, Patterns, Male, Adolescents

## Abstract

**Background:**

Clustering of multiple health-compromising behaviours is associated with an increased risk of various chronic diseases. There are few studies on patterns of clustering of multiple health-compromising behaviours in adolescents. Therefore, the aim of this study is to assess how six health-compromising behaviours, namely, low fruit consumption, high sweet consumption, less frequent tooth brushing, low physical activity, physical fighting and smoking, cluster among Saudi male adolescents.

**Methods:**

A representative stratified cluster random sample of 1,335 Saudi Arabian male adolescents living in Riyadh city answered a questionnaire on health-related behaviours. Hierarchical Agglomerative Cluster Analysis (HACA) was used to identify cluster solutions of the six health-compromising behaviours.

**Results:**

HACA suggested two broad and stable clusters for the six health-compromising behaviours. The first cluster included low fruit consumption, less frequent tooth brushing and low physical activity. The second cluster included high sweets consumption, smoking and physical fighting.

**Conclusions:**

The six health-compromising behaviours clustered into two conceptually distinct clusters among Saudi Arabian male adolescents, one reflecting non-adherence to preventive behaviours and the second undertaking of risk behaviours. Clustering of health behaviours has important implications for health promotion.

## Background

Heath-related behaviours such as smoking, alcohol misuse, physical inactivity and unhealthy diets contribute significantly to chronic diseases [1]. Many studies report on interrelationships between some health-related behaviours such as physical activity with healthy eating habits [2], and smoking with eating habits [3]. The interrelationships between health-related behaviours are considered to be multidimensional [4–6]. Roysamb et al. [7] suggested a multidimensional model consisting of three groups of behaviours, namely, “high action”, “addiction” and “protection” behaviours. Moreover, the Problem Behaviour Theory supports the view that the relationships between problem behaviours are multidimensional in nature [8]. The multidimensional approach assumes that certain health-related behaviours tend to cluster in a number of different patterns among both adolescents and adults [9–12]. For example, Raitakari et al. [13] found that a poor diet, smoking, physical inactivity and excessive consumption of alcohol clustered in young adults, while Neumark-Sztainer et al. [14] found associations between different health-compromising behaviours, namely, unhealthy weight loss, substance abuse, suicide risk, delinquency, and sexual activity. In an extensive systematic review of studies published between 1995 and 2003 to identify the clustering of four health-related behaviours (smoking, alcohol abuse, safe sex and healthy nutrition) in adolescents, Wiefferink et al. [15] identified three patterns of clustering. The largest cluster was adolescents who ate healthily, were not smokers and who did not drink alcohol. The second cluster was adolescents who ate unhealthily, smoked and drank alcohol. The third cluster comprised adolescents who ate unhealthily but did not smoke or drink alcohol. Later, Van Nieuwenhuijzen et al. [9] identified two clusters of behaviours for younger adolescents aged 12–15 years, and three clusters for adolescents aged 16–18 years.

Clustering is important because the co-occurrence of multiple health-compromising behaviours is associated with increased risk of chronic diseases including certain cancers and cardiovascular diseases [16]. The increased risk is the result of accumulation and synergistic adverse effects of behaviours on health [17]. Moreover, behavioural patterns in adulthood are primarily shaped during the adolescence period [18]. Therefore, understanding how health-related behaviours relate to one another in adolescents has important implications throughout the life course [19].

Different types of behaviours encompass different aspects of adolescents’ lifestyle. Behaviours related to healthy eating, oral hygiene practices, physical activity, physical fighting, and smoking have a considerable immediate and longer term effect on the health of adolescents and are related to one another. For example, higher fruit intake is associated with increased physical activity [20] and with lower rates of smoking and alcohol consumption [21]. In terms of dietary behaviours, lower fruit intake goes together with higher consumption of sweets and soft drinks and saturated fat [22]. Hygiene behaviour such as toothbrushing frequency, is linked to patterns of smoking [23]. Indeed, smoking is viewed as a “gateway behaviour” to other risky behaviours like drug use and drinking alcohol [24]. The Problem Behaviour Theory postulates that physical fighting is a reliable predictor of multiple risk behaviours such as carrying weapons, injury [25, 26], and substance abuse [27]. Despite these associations between different behaviours, research has generally focused on a limited number of behaviours at a time, with most studies looking at the clustering of two behaviours, thereby limiting understanding of the inter-relationships between different and diverse health-related behaviours among adolescents. Furthermore, these studies have employed basic statistical techniques that either assess only the associations between specific behaviours in a cluster or look at whether the prevalence of predetermined clusters of behaviours is higher than expected; these are correlation coefficients and observed/expected ratios, respectively. While useful, these techniques can only look at behavioural clusters that are predetermined, rather than explore whether the different behaviours form clusters according to theoretical expectations. To address these issues, this study sets out to provide useful insights into clustering and inter-relationships between a wide and diverse range of adolescents’ health-related behaviours. Therefore, the objective of this study was to assess how six health-compromising behaviours, namely, low fruit consumption, high sweet consumption, less frequent tooth brushing, low physical activity, physical fighting and smoking cluster together among male adolescents in Riyadh, Saudi Arabia.

## Methods

Subjects were Saudi males in two age groups: 13–14 year old students in 8^th^ grade intermediate schools and 17–19 year old students in 12^th^ grade secondary schools. These two age groups were considered to represent respectively the onset of physical and emotional changes in early adolescence, and later adolescence when young people are about to choose their future careers and have a greater degree of autonomy [28]. For practical local reasons, females could not be included in the study because all researchers were males, and men are not allowed to enter schools for girls in Saudi Arabia. Public and private schools for intermediate and secondary stages were selected. The samples were randomly selected from 515 intermediate and secondary schools in Riyadh. Schools for special needs children were excluded. Based on recommendations of the Health Behaviour in School-Aged Children (HBSC) international protocol, a cluster design was used [29]. The sampling frame was the list of schools for the whole Riyadh city. Stratified cluster random sampling was used to produce more precision and better representatives of the study population. The sampling frame was divided into four strata (public intermediate schools, public secondary schools, private intermediate schools, and private secondary schools). Schools were selected from each stratum by simple random sampling. As young and older adolescents were required for the study, all classes of only Grade 8 and Grade 12 in the selected schools were recruited. All students attending the selected classes on the day of the survey were invited to participate.

The sample size calculation was based on estimates of behavioural clustering from a pilot study and considered power of 80%, a = 0.05, a design factor of 1.2 to account for cluster sampling and 20% over-sampling for non-response. The calculated minimum final sample size was 980 students. For a representative sample of the relevant population in Riyadh, a self-weighting sample was used to select students from each stratum with the same proportion as in the general population [30]. That resulted in a sample size of 1100 students.

A self-administered classroom-based questionnaire used in the WHO cross-national study on Health Behaviour in School-Aged Children (HBSC) was adapted for use in this study [29]. The questionnaire included health-related behaviours, demographic characteristics, parent’s occupation and school environment. The questionnaire was developed in English and translated into Arabic by two qualified translators who were native speakers of Arabic and proficient in English. After that, the consensus Arabic questionnaire was backward translated into English and the backward translation was reviewed and compared for discrepancies with the original version [31]. No major differences were found. In addition, the Arabic questionnaire was reviewed by an expert teacher and then tested in a pilot study.

This study was approved by the University College London (UCL) Research Ethics Committee and the General Administration of Education at Riyadh Region, Saudi Arabia. Informed consent forms and information sheets were distributed through schools to parents and guardians. Positive parental written consent for all participants was received prior to the commencement of data collection. In conformity with procedures stipulated in the HBSC protocol [29], students were assured about anonymity and confidentiality of their responses. They were also given appropriate written and verbal instructions by the principal author (SA) at the beginning of the anonymised questionnaire.

### Measures

Dietary behaviours included weekly frequency of eating fruit and sweets (*never, less than once a week, once a week, 2–4 days a week, 5–6 days a week, once a day every day, more than once every day)*[29]. Tooth brushing frequency was reported as *“More than once a day, once a day, at least once a week but not daily, less than once a week, never”*[32]. Physical activity was assessed through the 60 minute Moderate-to-Vigorous Physical Activity (MVPA) measure [33]. Physical fight frequency in the past year reported as *“I have not been in a physical fight in the past 12 months”* to “*four times or more”*[34]. Smoking was measured by *“How often do you smoke tobacco at present?”* Response options ranged from: *“Every day” to “I do not smoke”*[29].

### Statistical analysis

The six health-related behaviours had different categorizations ranging from 4 to 7 categories. In order to make them directly comparable, they were dichotomized into binary variables (0 = healthy behaviour; and 1 = health-compromising behaviour) based on public health recommendations. Fruit consumption was dichotomized into once or more daily vs. less than once daily; sweet consumption into less than once daily vs. once or more daily; tooth brushing into twice or more daily vs. less than twice daily. For physical activity, an answer of 5 days or more per week indicates meeting physical activity recommendations, while less than 5 days per week indicates not meeting recommendations [33]. Physical fighting was categorised into none vs. one time or more in the last 12 months. Tobacco smoking was grouped into non-smoker and current smoker (at least once per week).

Pairwise correlations using Phi test for binary variables were used. Analysis of clustering was based on the Hierarchical Agglomerative Cluster Analysis (HACA). HACA is the most appropriate approach in identifying clusters of health-related behaviours [35–37]. It produces more stable cluster solutions compared to non-Hierarchical Cluster Analysis, and allows grouping of subjects that have similar characteristics across different variables leading to homogenous empirical types [35, 37]. Following guidance from the literature [37], the stability of the clusters was verified by repeating the HACA on different sub-samples drawn randomly from the study sample. The stability of the identified clusters is also essential for their validity. Furthermore, we also calculated the correlation coefficients between the different behaviours of each identified clusters as another approach to validate the identified cluster structures [36]. HACA was therefore used to identify stable cluster solutions for the multiple health-compromising behaviours, through an average linkage algorithm between groups that identified homogenous subgroups within the heterogeneous sample. We used Squared Euclidean distance as the measure of proximity, as it is suitable for binary variables [36]. The number of identifiable clusters was not known a priori. The Statistical Package for Social Sciences (SPSS for Windows, version 16.0/PC; SPSS, Chicago, Illinois, USA) was used for statistical analysis.

## Results

Of the 515 schools in Riyadh, 22 were randomly selected and agreed to participate in the study. We invited 1,354 eligible students to participate. There were no refusals by students or parents, but 19 questionnaires were excluded from the analysis because they were not fully completed. Therefore, the analytical sample was 1,335 students.

More than half the sample (54%) were 17–19 years old, and 52% of the adolescents attended public schools. About 85% of adolescents ate fruit less than once daily, 74% brushed their teeth less than twice daily, 64% had low physical activity, 51% had been involved in physical fighting at least once or more in the last 12 months, 43% ate sweets once or more daily and 23% smoked tobacco (Table 1). Low fruit consumption was positively correlated with low physical activity and less frequent tooth brushing (p < 0.01). Smoking was positively correlated with physical fighting and high sweet consumption (p < 0.01) (Table 2).

**Table 1:** **Characteristics of study sample**

	n	%
**Age**		
13-14 years	613	45.9
17-19 years	722	54.1
**School type**		
Private	640	47.9
Public	695	52.1
**Health-compromising behaviours**		
Low fruit consumption (Less than once daily)	1130	84.6
High sweet consumption (Once or more daily)	579	43.4
Less frequent toothbrushing (Less than twice daily)	991	74.2
Low physical activity (Less than 5 days per week of MVPA)	850	63.7
Physical fighting (One time or more per year)	677	50.7
Smoking (At least once or more per week)	312	23.4

**Table 2:** **Pairwise correlations between health-compromising behaviours**

	Low fruit consumption	High sweet consumption	Less frequent toothbrushing	Low physical activity	Physical fighting	Smoking
Low fruit consumption	1					
High sweet consumption	-0.03	1				
Less frequent toothbrushing	0.08**	0.03	1			
Low physical activity	0.12**	-0.01	0.03	1		
Physical fighting	-0.002	0.02	-0.03	-0.03	1	
Smoking	-0.004	0.08**	0.03	0.11**	0.08**	1

**Phi correlation was significant p < 0.001.

**Figure 1:**
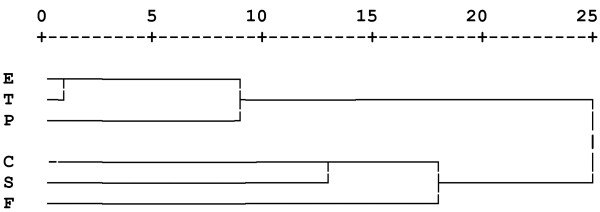
**Tree diagram of hierarchical agglomerative cluster analysis of the six health-related behaviours.** The dendrogram provides a visual presentation of the distance at which clusters are combined. It is read from left to right and the vertical lines show joined clusters. The position of the vertical line on the scale indicates the distance at which clusters are joined. Variables with smaller distance have higher homogeneity and they are combined with a vertical line linking them in a cluster, while the variables with larger distance indicate the least homogenous clusters. The distances (agglomeration coefficients) displayed in the top of the plot are rescaled (by default) to fall into a range of 1 to 25.

Figure 1 shows the hierarchical tree plot (dendrogram) which is a visual presentation of the distance (agglomeration schedules) at which clusters are combined. Pairs of variables with smaller distances were more similar and were combined with average linkage in a group, while the variables with larger distances indicate the least homogenous groups [36]. Based on the proximity coefficients, low fruit consumption (E) and less frequent tooth brushing (T) were combined together in one group. After that, low physical activity (P) was also combined with E and T to form a cluster (Cluster 1). In the third stage, high sweet consumption (C) and smoking (S) formed a new group. In the fourth stage, physical fighting (F) combined with C and S to form a new cluster (Cluster 2). At stage four, there were two distinct clusters, with large distances (agglomeration coefficients) between them, thereby representing the best solution for this study population. These two distinct clusters with different patterns of health-compromising behaviours collectively included all six health-related behaviours.

The first cluster at the top of the dendrogram plot consisted of low fruit consumption, less frequent tooth brushing and low physical activity. The second cluster at the bottom of the dendrogram plot included high sweet consumption, smoking and physical fighting. The stability and validity of the clusters was confirmed by repeating the HACA on different sub-samples drawn randomly from the study sample. Also, significant associations between the variables in each cluster validated the cluster structure (Table 2).

## Discussion

The HACA analysis identified two broad and stable clusters of health-compromising behaviours. The first cluster included low fruit consumption, less frequent tooth brushing and low physical activity, and the second cluster included high sweets consumption, smoking and physical fighting. These two clusters are quite distinct conceptually, with the first reflecting non-adherence to preventive behaviours, while the second, to undertaking risk behaviours. Previous studies reported associations between low fruit and vegetables consumption and low physical activity in adolescence [9, 38–41], and between tooth brushing and eating habits [42]. However, those studies only reported associations between two behaviours at a time, and did not look at clustering patterns of multiple health-related behaviours. Our findings go further in terms of identifying distinct clusters of multiple health-related behaviours. For example, we showed that less frequent tooth brushing clustered with low fruit consumption and low physical activity.

The second cluster (high sweets consumption, smoking and physical fighting) agrees partly with a systematic review that reported a significant association between high sweet consumption and smoking [21], while another study showed significant association between substance abuse and fighting [27]. The above-mentioned studies only reported associations between two behaviours. One potential explanation for our results showing a cluster of high sweet consumption, smoking and physical fighting is that these behaviours may have determinants in common [43]. For example, delinquency and rebellious behaviours might be important risk factors in adolescents, especially for clustering of smoking with physical fighting [9].

Previous research indicated that health-related behaviours were not independent of each other [5] and their interrelationships were multidimensional [4, 44]. Furthermore, the present study used statistical methods, the HACA, not used heretofore to assess clustering of health-related behaviours. The HACA is a rigorous methodological tool that can be used to highlight the multidimensional relationships between health-related behaviours. It gives more stable cluster solutions compared to non-Hierarchical Cluster Analysis. Though it was used here in an exploratory manner, it has been used extensively in other research fields [37]. Our results confirmed that HACA is a valuable method to identify clustering of health-related behaviours.

This is the first study on the prevalence and clustering of multiple health-related behaviours among a representative sample of Saudi Arabian male adolescents. We used established data collection tools, adapted from the HBSC [29], and had a very high response rate due to excellent cooperation from the adolescents in the selected schools. Moreover, a wide variety of important health-related behaviours among adolescents were included. However, this study has certain limitations. It was conducted only in Riyadh city, which might explain the relatively homogeneous study population. Also, for reasons beyond our control, girls were not included in this study. The data are self–reported, therefore might be subject to recall and social desirability bias. However, previous research showed that confidentiality and anonymity of self-reports reduces bias and provides reliable and valid data [45]. The six health-related behaviours were dichotomized which might lead to loss of some information about individual differences [46]. As the health-related behaviours included in this study had different scales and categories, dichotomization based on public health recommendations was considered appropriate to assess clustering of multiple health-related behaviours with same metric.

Our results have important implications for public health practice. Showing that there are two distinct and broad clusters of health-compromising behaviours emphasizes the importance of a cluster-based approach in health promotion intervention planning and the potential greater impact of targeting multiple health-related behaviours [47, 48]. Oral health-related behaviours were clustered with general health-related behaviours. That emphasizes the importance of multidisciplinary health promotion interventions using the Common Risk Factor Approach [49].

## Conclusions

The six health-compromising behaviours (low fruit consumption, high sweet consumption, less frequent tooth brushing, low physical activity, physical fighting and smoking) clustered into two clusters. One cluster contained health-compromising behaviours; not conforming to preventive behaviours for fruit consumption, physical activity and tooth brushing. The other cluster consisted of risk-taking behaviours such as smoking, physical fighting and high sweets consumption. These two stable clusters appear to be representative clusters among Saudi Arabian male adolescents in Riyadh city.

## Abbreviations

HACA: Hierarchical Agglomerative Cluster Analysis
HBSC: Health Behaviour in School-Aged Children
MVPA: Moderate-to-Vigorous Physical Activity
E: Low fruit consumption
T: Less frequent tooth brushing
P: Low physical activity
C: High sweet consumption
S: Smoking
F: Physical fighting.
